# One-Pot Synthesis of Ultra-Small Pt Nanoparticles-Loaded Nitrogen-Doped Mesoporous Carbon Nanotube for Efficient Catalytic Reaction

**DOI:** 10.3390/nano13192633

**Published:** 2023-09-25

**Authors:** Qian Zhang, Minying Wu, Yuanyuan Fang, Chao Deng, Hsin-Hui Shen, Yi Tang, Yajun Wang

**Affiliations:** 1 Department of Chemistry, Shanghai Key Laboratory of Molecular Catalysis and Innovative Materials, and Laboratory of Advanced Materials, Fudan University, Shanghai 200438, China; 2 College of Chemistry & Materials Engineering, Wenzhou University, Wenzhou 325027, China; 3 Department of Materials Science and Engineering, Monash University, Clayton, VIC 3800, Australia

**Keywords:** platinum, nitrogen doping, mesoporous carbon nanotubes, catalysis

## Abstract

In this study, Pt nanoparticles-loaded nitrogen-doped mesoporous carbon nanotube (Pt/NMCT) was successfully synthesized through a polydopamine-mediated “one-pot” co-deposition strategy. The Pt source was introduced during the co-deposition of polydopamine and silica on the surface of SiO_2_ nanowire (SiO_2_ NW), and Pt atoms were fixed in the skeleton by the chelation of polydopamine. Thus, in the subsequent calcination process in nitrogen atmosphere, the growth and agglomeration of Pt nanoparticles were effectively restricted, achieving the in situ loading of uniformly dispersed, ultra-small (~2 nm) Pt nanoparticles. The method is mild, convenient, and does not require additional surfactants, reducing agents, or stabilizers. At the same time, the use of the dual silica templates (SiO_2_ NW and the co-deposited silica nanoclusters) brought about a hierarchical pore structure with a high specific surface area (620 m^2^ g^−1^) and a large pore volume (1.46 cm^3^ g^−1^). The loading process of Pt was studied by analyzing the electron microscope and X-ray photoelectron spectroscopy of the intermediate products. The catalytic performance of Pt/NMCT was investigated in the reduction of 4-nitrophenol. The Pt/NMCT with a hierarchical pore structure had an apparent reaction rate constant of 0.184 min^−1^, significantly higher than that of the sample, without the removal of the silica templates to generate the hierarchical porosity (0.017 min^−1^). This work provides an outstanding contribution to the design of supported noble metal catalysts and also highlights the importance of the hierarchical pore structure for catalytic activity.

## 1. Introduction

Pt catalysts have important applications in many kinds of reactions, especially reduction reactions, because of their high activity, selectivity, and stability [1,2,3]. The catalytic efficiency of Pt nanoparticles supported on a substrate can be significantly enhanced due to their small size, homogeneous dispersion, expansive active surface area, and resistance to sintering both during preparation and catalytic reactions. Moreover, the interaction between Pt species and supportive materials is advantageous for regulating the electronic configuration of Pt [4,5,6,7]. In the past few decades, because carbon-based carriers usually have a high surface area, large pore volume, chemical stability, and thermal conductivity, carbon-supported metal catalysts have gained a lot of attention [8,9,10,11]. However, the microporous structure of commonly used carbon supports restricts the transport and diffusion of reaction molecules to some extent; at the same time, the anchorable site used to supply the active phase is insufficient, which leads to the uneven dispersion of metal nanoparticles on the carbon surface and may cause further aggregation and leaching. Therefore, it is urgent to develop porous carbon materials with open pores and more supporting sites to prepare metal/carbon catalysts to expand their application prospects.

Researchers have developed porous carbon materials doped with heteroatoms (B, N, S, etc.) [12,13,14,15]. The doping of heteroatoms in carbon materials is able to alter the surface properties, thereby broadening their potential application range. Among the various dopants, nitrogen has been widely studied. Previous work shows that nitrogen is conducive to improving the catalytic performance of heterogeneous catalytic reactions of metal/nitrogen-doped carbon catalysts [16,17,18]. The doped nitrogen can change the acid-base substance on the surface of the carrier, increase the electron interaction between the metal and the carrier, and accelerate the electron transfer in the catalytic system [19].

In order to realize the loading of platinum nanoparticles, the commonly used method is post-loading, that is, depositing metal precursors on pre-synthesized nitrogen-doped carbon materials through traditional methods such as impregnation and deposition precipitation [20]. This usually requires the addition of reducing agents like sodium borohydride and ethylene glycol [21,22]. And, for smaller and highly dispersed metal nanoparticles, it is often necessary to add certain stabilizers, such as citric acid and polyvinylpyrrolidone [23,24]. In recent years, a more convenient, “one-pot” co-deposition method has been developed for the in situ deposition of metal nanoparticles on the functionalized surface with amino groups, polydopamine (PDA), and others, avoiding the use of stabilizers [25,26,27,28].

Recently, we reported a method for the preparation of nitrogen-doped mesoporous carbon nanotubes (NMCTs) through the co-deposition of PDA and silica on a sacrificial silica nanowire (SiO_2_ NW) [29]. Herein, we extended this “dual-silica template” strategy to the synthesis of ultra-small Pt nanoparticles-loaded N-doped mesoporous carbon nanotubes (Pt/NMCT) by combing the PDA-mediated one-pot co-deposition approach. The chloroplatinic acid (H_2_PtCl_6_, platinum source) introduced in the “co-deposition” step was fixed by the chelation of the amino group of PDA. During the subsequent carbonization process, the overgrowth of the Pt nanoparticles in the PDA skeleton was restricted, thus achieving the in situ loading of the ultra-small and uniformly dispersed Pt nanoparticles without the need for additional protective agents or reducing agents. The prepared Pt/NMCT had a high specific surface area, a large porosity, a hierarchical pore structure, and abundant nitrogen-doping. The loaded Pt nanoparticles had a uniform particle size (ca. 2 nm), and the content of Pt was 9.5 wt%. The chemical structure and morphological changes in Pt in the process of material synthesis were studied. Also, the catalytic performance was evaluated through the reduction reaction of 4-nitrophenol (4-NP) to 4-aminophenol (4-AP).

## 2. Materials and Methods

### 2.1. Materials

Dopamine hydrochloride (DA·HCl, 98%), tetraethoxysilane (TEOS, A.R.), and chloroplatinic acid (H_2_PtCl_6_·6H_2_O, ≥37.50% Pt basis) were provided from Sigma-Aldrich. Hydrofluoric acid (40 wt%), ammonium hydroxide solution (28 wt%), ethanol (EtOH, A.R.), and sodium borohydride (A.R.) were purchased from Sinopharm Chemical Reagent. 4-nitrophenol (4-NP, A.R.) was purchased from Aladdin. The water used in the experiment was ultrapure water (18.2 MΩ cm^−1^).

### 2.2. Synthesis of Pt/NMCT

The monodispersed SiO_2_ NW was prepared according to the literature [30]. A total of 50 mg of SiO_2_ NW was fully dispersed in a mixture of 30 mL of EtOH, 4 mL of water, and 2.5 mL of concentrated ammonia. A total of 0.9 mL of a 100 mg mL^−1^ DA aqueous solution was added while stirring. Forty-five minutes later, 50 μL of 0.1 g mL^−1^ H_2_PtCl_6_ solution and 100 μL of TEOS were added, and the solution was then stirred for 4 h. After centrifugation and washing three times, the sediment was dried at 70 °C in an oven. The obtained powder (SiO_2_ NW@Pt/PDA-SiO_2_) was then calcined at 300 °C and 800 °C for 2 h under nitrogen atmosphere. The calcined samples (SiO_2_ NW@Pt/C-SiO_2_) were dispersed into a 5.0 M HF solution to remove SiO_2_. The obtained product was named Pt/NMCT. For comparison, Pt nanoparticles-loaded mesoporous carbon sphere (Pt/NMCS) was also synthesized by the same experimental method as Pt/NMCT, except that no SiO_2_ NW template was added.

### 2.3. Catalytic Reduction Reaction of 4-NP

A total of 3 mL of 0.1 mM 4-NP aqueous solution (light yellow color) was taken, and 200 μL of newly prepared 0.1 M NaBH_4_ solution was added, and the solution turned a bright yellow color. After that, 20 μL of catalyst dispersion (48 μg mL^−1^ Pt) was quickly added, and the change in the absorption peak at 400 nm was monitored at one-minute intervals using a UV-Vis spectrophotometer.

### 2.4. Characterization

The morphology of the samples was observed using a scanning electron microscope (SEM, Ultra 55, Carl Zeiss, Jena, Germany) at 5 kV, a Hitachi HT7800 transmission electron microscope (TEM, Tokyo, Japan) operating at 100 kV, and a FEI Tecnai G2 F20 S-Twin TEM (Hillsboro, OR, USA) operating at 200 kV. The element distribution and content were obtained by an energy-dispersive X-ray spectrometer (EDS, FEI, Hillsboro, OR, USA) and an inductively coupled plasma-optical emission spectrometer (ICP-OES, PerkinElmer Optima 8000, Waltham, MA, USA). The phase information was evaluated by a Bruker AXS D2 PHASER diffractometer (Karlsruhe, Germany) with a tube voltage of 5 kV and a current of 30 mA (Cu-K, λ = 1.54056 Å). N_2_ sorption analysis was performed on a Quadrasorb apparatus (Quantachrome Instruments, Boynton Beach, FL, USA). The specific surface area was calculated by the Brunauer–Emmett–Teller (BET) method. Pore volumes were determined at *p*/*p*_0_ = 0.989. Pore size distributions were acquired using the quenched solid density functional theory (DFT) model with the desorption branch. UV-vis spectra were obtained with a Shimadzu UV-2600 spectrophotometer (Kyoto, Japan). A Kratos Axis Ultra DLD spectrometer (Manchester, UK) was engaged to obtain the X-ray photoelectron spectroscopy (XPS) spectra. The binding energies in the XPS spectra were adjusted for specimen charging with reference to C 1s at 284.6 eV.

## 3. Results and Discussion

**Figure 1:**
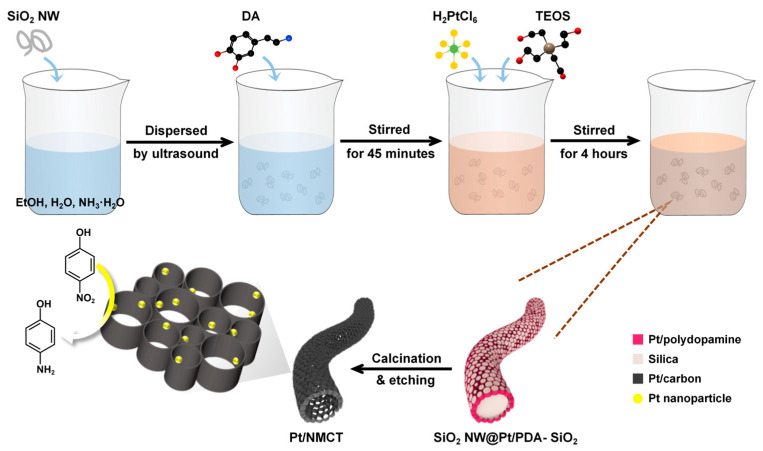
Schematic illustration for the synthesis of the Pt nanoparticles loaded-N-doped mesoporous carbon nanotube (Pt/NMCT).

**Figure 2:**
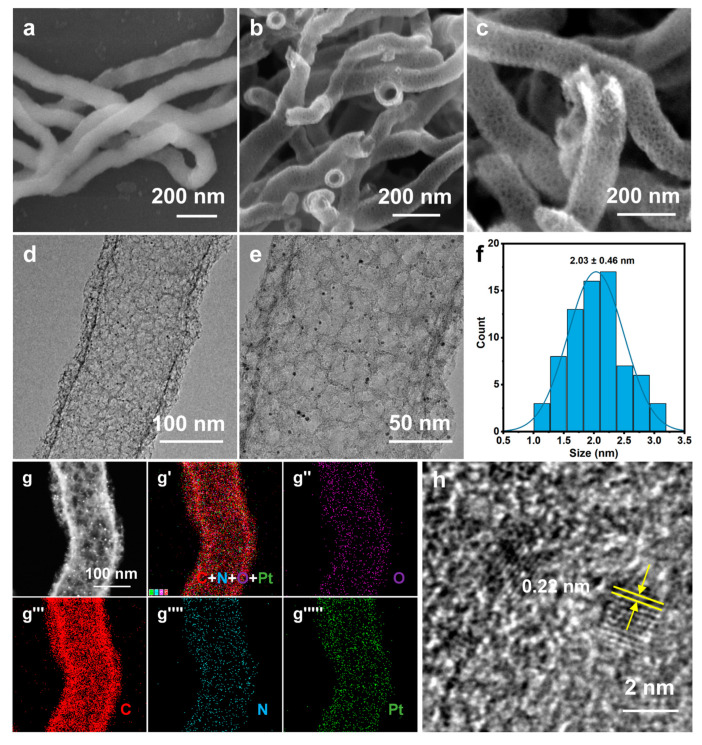
(**a**) SEM image of SiO_2_ NW; (**b**,**c**) SEM images, (**d**,**e**) TEM images, (**f**) Pt particle size distribution, (**g**) HAADF-STEM image, (**g′**–**g′′′′′**) EDS elemental mappings, and (**h**) high-resolution TEM image of Pt/NMCT.

As shown in Figure 1, Pt/NMCT is prepared by a polydopamine-mediated “one-pot” co-deposition method, followed by carbonization and removal of the silica template. To start with, the DA in the reaction solution was self-polymerized and sequestered the Pt species from the solution. The SiO_2_ NW used in synthesis is shown in Figure 2a. The formed PDA oligomers and SiO_2_ nanoparticles (from the hydrolysis of added TEOS) were co-deposited on the surface of SiO_2_ NW, producing a core-shell structured SiO_2_ NW with a co-deposited SiO_2_ and Pt-loaded PDA shell. During the following carbonization procedure, the PDA-loaded Pt was simultaneously reduced by utilizing the reducibility of PDA. After removal of the co-deposited SiO_2_ nanoparticles and the SiO_2_ NW, hierarchically porous Pt/NMCT was obtained.

The electron microscopy (SEM/TEM) was used to characterize the morphology of Pt/NMCT. According to the SEM images (Figure 2b,c), Pt/NMCT has a hollow tubular structure and a mesoporous tube wall. The TEM image (Figure 2d) indicates that the Pt nanoparticles loaded in the NMCT are evenly distributed and uniform in particle size. The statistics of the particle size from the TEM image (Figure 2e) show that the particle size is about 2 nm (Figure 2f). The HAADF-STEM image (Figure 2g) further shows that Pt nanoparticles have a small size, a uniform size distribution, and good dispersion throughout the carrier NMCT. The EDS elemental mappings (Figure 2g′–g′′′′′) demonstrate that the N and Pt elements are uniformly distributed in Pt/NMCT. The content of Pt was measured to be 9.5 wt% by ICP-OES. From Figure 2h, the lattice fringe of Pt nanoparticles can be seen in the HRTEM image with a crystal plane spacing of 0.22 nm, which is consistent with the (111) crystal plane of Pt [31].

**Figure 3:**
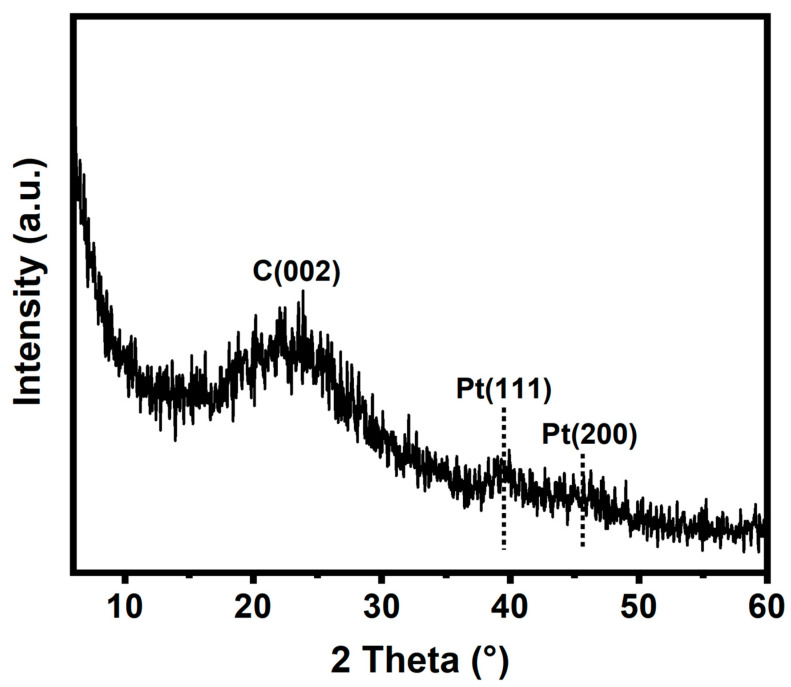
XRD pattern of Pt/NMCT.

The crystal structure of Pt/NMCT was characterized by XRD. In the XRD pattern of Pt/NMCT (Figure 3), the material exhibits diffraction peaks at around 39.8° and 46.2°, corresponding to the (111) and (200) crystalline planes of Pt, respectively [32]. The weakening and broadening of the XRD diffraction peak also confirm the small particle size of Pt nanoparticles [20]. The wide diffraction peak near 23° is due to partially graphitized carbon [33].

**Figure 4:**
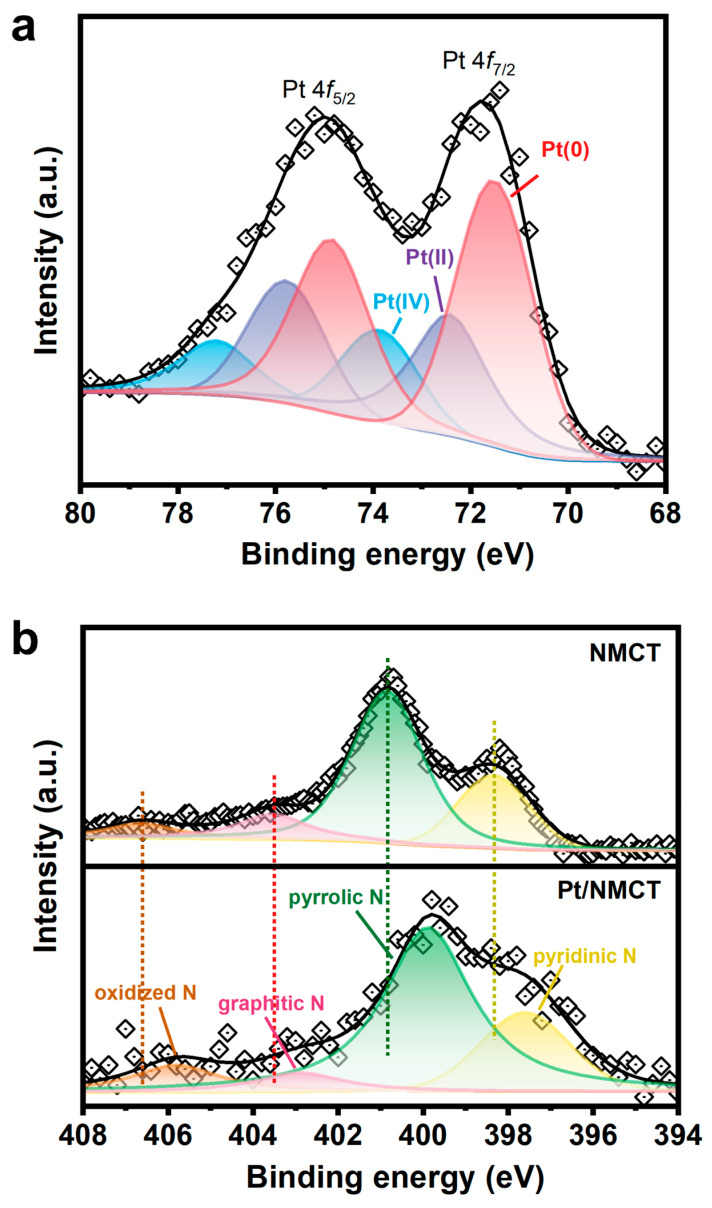
(**a**) Pt 4f high-resolution X-ray photoelectron diffraction spectra (XPS) of Pt/NMCT; (**b**) N 1s XPS of NMCT and Pt/NMCT.

XPS was applied to investigate the chemical states of Pt/NMCT. The Pt 4f XPS spectrum of Pt/NMCT (Figure 4a) was subjected to peak fitting, and three groups of 4f split peaks (4f_7/2_ and 4f_5/2_) were obtained. The peaks of 4f_7/2_ are located at 71.5, 72.4 and 73.9 eV, respectively, corresponding to Pt at 0, +2 and +4 valence states [20,34]. This shows that most Pt is reduced, accounting for more than half of the total Pt content. The N 1s spectrum (Figure 4b) of Pt/NMCT can be divided into four peaks centered at 397.6, 399.9, 402.9 and 405.8 eV, assigned to pyridinic N, pyrrolic N, graphitic N, and oxidized N, respectively [35,36]. The relative contributions of pyridinic N, pyrrolic N, graphitic N, and oxidized N are 21.7, 60.8, 7.3 and 10.2%, respectively, demonstrating the successful doping of N atoms in the carbon skeleton. Compared with the N 1s spectrum of NMCT synthesized in the absence of a Pt source, the peak in the N 1s spectrum moves in the direction of low binding energy, which indicates a strong interaction between Pt nanoparticles and the N atom-containing surface of NMCT [33]. The interaction between the empty orbitals of Pt atoms and the free electron pairs of N atoms (such as pyridinic N) can limit the mobility of Pt nanoparticles and prevent them from aggregation, enhancing their durability and stability [23]. In addition, the sp^2^-hybridized N atoms that supply electrons to the delocalized π bond in carbon materials (such as pyridinic N and graphitic N) can modify the electronic structure of Pt nanoparticles or enhance the adsorption of 4-NP, thereby improving the intrinsic catalytic activity of the catalyst [37,38].

**Figure 5:**
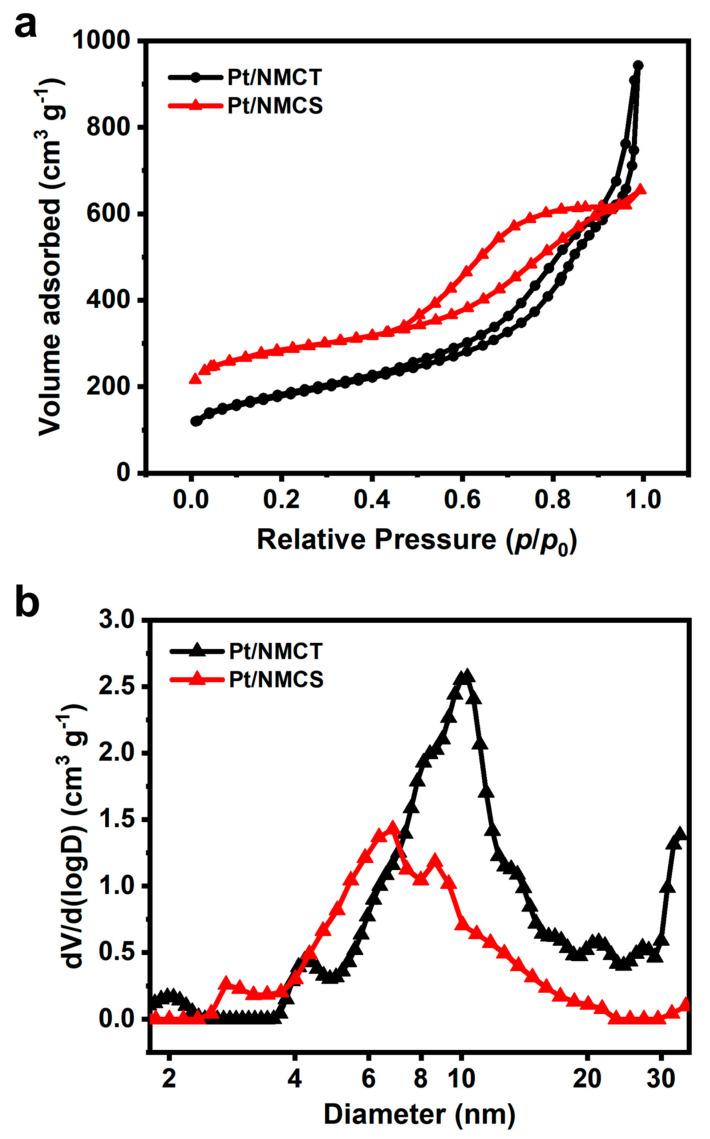
(**a**) N_2_ sorption isotherms and (**b**) pore size distribution of Pt/NMCT.

The texture property of the Pt/NMCT was studied through the low-temperature nitrogen gas adsorption experiment, and the isotherms and pore size distribution are shown in Figure 5. The nitrogen adsorption/desorption isotherms can be classified as type IV with an H4 hysteresis loop according to the IUPAC classification. This shows that Pt/NMCT has a hierarchical pore structure with micropores, mesopores, and macropores, and the pore distribution curve (Figure 5b) also verifies this conclusion. From the pore size distribution plot, the mesopores are mainly distributed in the range of 4–20 nm. The mesopores are from co-deposited silica, and the macropores are mainly from the hollow tube structure brought about by the SiO_2_ NW template. Based on the calculation, the specific surface area of the material is 620 m^2^ g^−1^, and the total pore volume is 1.46 cm^3^ g^−1^. The high surface area and pore volume of the sample are conducive to the diffusion of molecules, the thorough dispersion of Pt nanoparticles, and the exposure of active sites, which are beneficial for improved catalyst activity.

In order to monitor the Pt loading and reduction process, we also analyzed the intermediates SiO_2_ NW@Pt/PDA-SiO_2_ and SiO_2_ NW@Pt/C-SiO_2_ by the SEM, TEM, and XPS techniques. A growth mechanism for Pt nanoparticles was also speculated.

**Figure 6:**
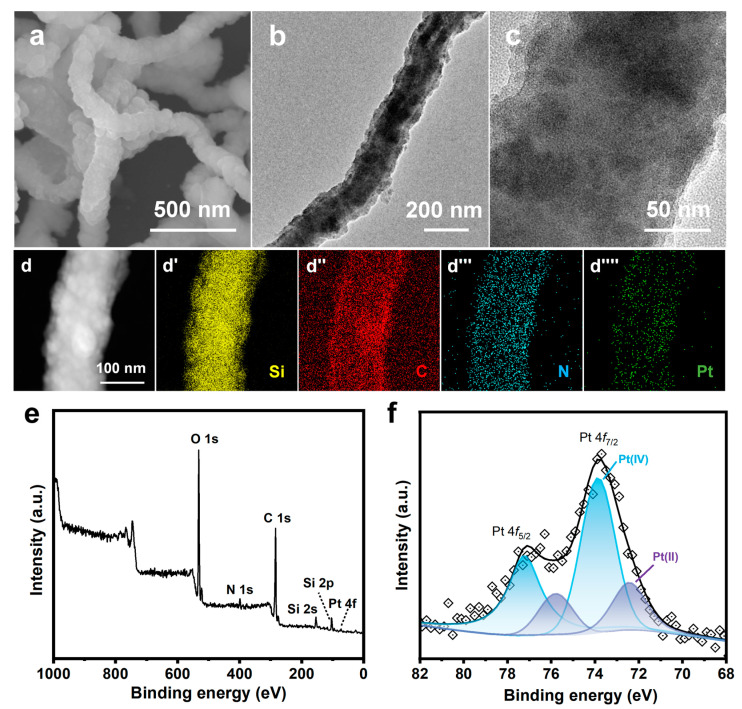
(**a**) SEM image, (**b**,**c**) TEM images, (**d**) HAADF-STEM image, (**d′**–**d′′′′**) EDS elemental mappings, (**e**) XPS full spectrum of SiO_2_ NW@Pt/PDA-SiO_2_, and (**f**) Pt 4f XPS high-resolution spectrum of SiO_2_ NW@Pt/PDA-SiO_2_.

There are no observable metal particles in SEM and TEM images of the intermediate SiO_2_ NW@Pt/PDA-SiO_2_ prepared in the co-deposition step (Figure 6a–c). From the EDS elemental mapping (Figure 6d) and the XPS full spectrum (Figure 6e), the loading of the Pt element can be clearly confirmed. In the XPS high-resolution spectrum of Pt 4f (Figure 6f), it can be seen that the main peak position of Pt is at a higher binding energy. After deconvolution of the 4f peaks, they are rearranged into two groups of peaks. The two 4f_7/2_ located at 72.4 eV and 73.9 eV, corresponding to the +2 and +4 valent, account for 26.2% and 73.7%, respectively. In other words, the Pt element in SiO_2_ NW@Pt/PDA-SiO_2_ exists in its ionic state. To demonstrate the interactions between PDA and Pt species during the co-deposition step, a simulated adsorption experiment was performed. In this simulated experiment, the PDA nanoparticles, synthesized under the same conditions except for the addition of SiO_2_ NW and TEOS, were suspended in a H_2_PtCl_6_ solution of an equivalent concentration to that in the co-deposition process. EDS analyses of the sediment revealed that PDA adsorbed a certain amount of Pt, probably through electrostatic and coordination interactions with PDA [39,40,41,42].

**Figure 7:**
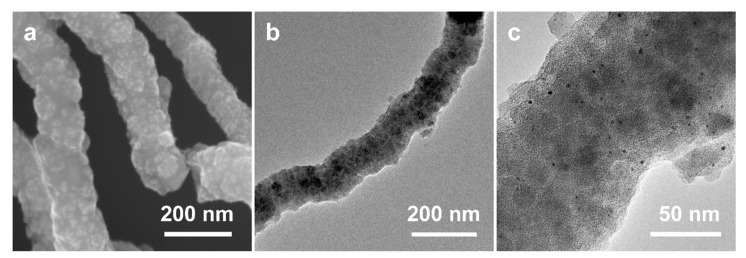
(**a**) SEM image and (**b**,**c**) TEM images of SiO_2_ NW@Pt/C-SiO_2_.

After the high-temperature calcination of SiO_2_ NW@Pt/PDA-SiO_2_ in a nitrogen atmosphere, it was transformed into SiO_2_ NW@Pt/C-SiO_2_ along with PDA carbonization. The SiO_2_ NW@Pt/C-SiO_2_ has a relatively rough surface without an obvious pore structure (Figure 7a,b), which does not seem to be very different from the SiO_2_ NW@Pt/PDA-SiO_2_. However, the presence of metal particles can be clearly seen in the TEM image of SiO_2_ NW@Pt/C-SiO_2_ (Figure 7c), which is significantly different from SiO_2_ NW@Pt/PDA-SiO_2_. After the subsequent HF etching, Pt/NMCT was obtained. Comparing its SEM and TEM images (Figure 2c,e) with those of SiO_2_ NW@Pt/C-SiO_2_ (Figure 7a,c), it can be found that the lumpy species in the SEM image of SiO_2_ NW@Pt/C-SiO_2_ (the patchy, slightly dark part in the TEM image) has been removed, leaving behind a honeycombed carbon skeleton structure. In addition, by comparing the TEM images of SiO_2_ NW@Pt/C-SiO_2_ (Figure 7b) and Pt/NMCT (Figure 2d), we can see that the original SiO_2_ NW was removed and formed a hollow tubular structure. Based on these results, the function of the dual-silica template agents was verified, namely that the co-deposited silica nanoclusters were used as mesoporous template agents, while the SiO_2_ NW acted as a sacrificial template for the formation of the tubular structure. In addition, from the TEM images of Pt/NMCT (Figure 2d,e), it can be seen that the Pt nanoparticles formed during the carbonization process are well-retained during the silica template etching process. To further prove the impact of the chelation and spatial restriction of the PDA-SiO_2_ composite on the growth of Pt nanoparticles, a comparison experiment was conducted by loading a similar content of Pt species onto a pre-prepared NMCT using rotary evaporation before calcination at the same temperature program used in the Pt/NMCT synthesis. The NMCT was synthesized by the same process as Pt/NMCT, except no H_2_PtCl_6_ was added. The resulting material exhibited a noticeable sintering of Pt nanoparticles of about 22.6 nm (Figure S1a) and very large Pt particles with a particle size of up to 200 nm (Figure S1b). These results demonstrated that the interaction between Pt and PDA and the confinement of Pt species in the PDA-SiO_2_ skeleton are important to suppress the overgrowth of the Pt nanoparticles in the calcination process [43,44].

Based on the above results, we speculated that the Pt nanoparticles undergo the following growth mechanism. With the addition of H_2_PtCl_6_ in the co-deposition step, the Pt is chelated by the amino group of dopamine and then evenly distributed in the composite shell. In the subsequent calcination process, the Pt is reduced in situ in the carbonization process of PDA, and the growth in reduced Pt nanoparticles was spatially restricted. After removal of the silica template, Pt/NMCT with enriched meso/macroporosity and uniformly distributed Pt nanoparticles is obtained.

To demonstrate the advantage of hollow structure in Pt/NMCT, a control sample of Pt nanoparticle-loaded nitrogen-doped carbon nanospheres (Pt/NMCS) was prepared without adding the SiO_2_ NWs as a template. As shown in Figure S2, the particles of Pt/NMCSs are highly agglomerated, and their mesopores are loaded with abundant Pt nanoparticles. The N_2_ sorption isotherms and pore size distribution curves (Figure 5) show that Pt/NMCS has less macroporosity and smaller mesopores than Pt/NMCT. Additionally, Pt/NMCS has a smaller pore volume (1.01 cm^3^ g^−1^) but a larger BET specific surface area (1032.6 m^2^ g^−1^) than Pt/NMCT due to the smaller mesopores and more abundant micropores that exist in Pt/NMCS.

**Figure 8:**
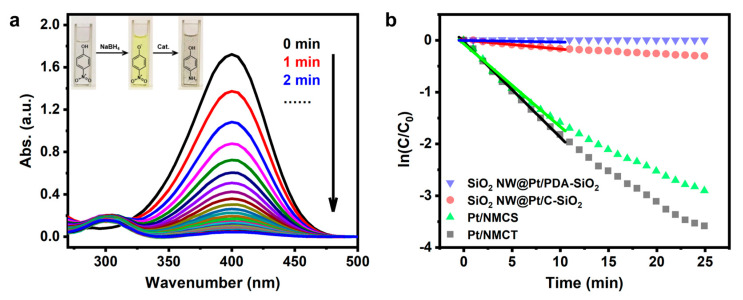
(**a**) UV-vis absorption spectra at different reaction times for the reduction of 4-nitrophenol using Pt/NMCT as the catalyst; (**b**) The curves of ln(*c*_t_/*c*_0_) versus time *t* using Pt/NMCT, Pt/NMCS, SiO_2_ NW@Pt/C-SiO_2_, and SiO_2_ NW@Pt/PDA-SiO_2_ as catalysts. Reaction conditions: [4-NP] = 0.093 mM, [NaBH_4_] = 6.2 mM, *T* = 25 °C, [Pt] = 0.30 mg L^−1^.

As a proof of concept, we evaluated the catalytic performance of Pt/NMCT in the reduction reaction of 4-NP. The 4-NP solution, after adding freshly prepared NaBH_4_ solution, showed a bright yellow color, due to the emergence of 4-nitrophenol ions induced by NaBH_4_ in an alkaline environment (Figure 8a-inset) [45]. When Pt/NMCT was added to the solution containing 4-NP and NaBH_4_, the yellow color gradually diminished until it became colorless. The reaction’s progress could also be quantitatively monitored by UV-vis spectroscopy. With the extension of reaction time, the peak intensity of 4-NP at 400 nm gradually decreases and finally disappears (Figure 8a). Meanwhile, a new absorption peak of 4-AP appears near 300 nm, indicating that the weakening of the peak is due to the reduction of 4-NP to 4-AP rather than the adsorption of 4-NP on the surface of the material [31,46].

In addition, the kinetics of the 4-NP reduction reaction were further studied. As the concentration of NaBH_4_ is much higher than that of the reactant (4-NP) in this study, the concentration of NaBH_4_ can be considered to be constant under reaction conditions, and the catalytic reaction follows pseudo first-order reaction kinetics [47]. The reaction rate of pseudo-first-order kinetics can be determined according to Equation (1) [25]:

ln (*c_t_*/*c*_0_) = −*kt* (1)

where *k* is the apparent rate constant in min^−1^ unit, and *c*_0_ and *c_t_* are the initial concentration and concentration of 4-NP at time *t*, respectively. As shown in Figure 8b (black square), ln(*c_t_*/*c*_0_) has a good linear correlation with the reaction time *t*, indicating quasi-first-order kinetics. The apparent rate constant *k* calculated from the slope of the fitting line is 0.184 min^−1^, which is similar to that of some promising Pt nanoparticles-supported catalysts in the literature [25,48,49].

In order to further illustrate the structural advantages of the materials prepared by the dual-silica template method and the necessary calcination to obtain active Pt species, we also investigated the performance of the control catalysts in the 4-NP reduction reaction, i.e., Pt/NMCS, the silica-retained sample SiO_2_ NW@Pt/C-SiO_2_, and the uncalcined sample SiO_2_ NW@Pt/PDA-SiO_2_. The curve between ln(*c*_t_/*c*_0_) and *t* obtained from UV-vis spectra is shown in Figure 8b. By calculating the slopes of the fitting curves, the apparent rate constants of SiO_2_ NW@Pt/PDA-SiO_2_, SiO_2_ NW@Pt/C-SiO_2_ and Pt/NMCS are 0.003, 0.017 and 0.160 min^−1^, respectively. The catalytic performance of Pt/NMCS is slightly worse than that of Pt/NMCT (0.184 min^−1^). Although Pt/NMCS has a higher surface area, which is normally beneficial to improving the catalyst activity, the longer diffusion pathway and the lack of macropores in Pt/NMCS may restrict the diffusion of guest molecules, thereby causing the inferior activity of Pt/NMCS [50]. Also, SiO_2_ NW@Pt/C-SiO_2_ shows significantly lower catalytic activity than Pt/NMCT due to the less porous structure of SiO_2_ NW@Pt/C-SiO_2_ without removal of the silica template. The worst activity was found in SiO_2_ NW@Pt/PDA-SiO_2_, which suggests that oxidized Pt is less active than the reduced one in this reaction.

The above results suggest that the Pt nanoparticles are mainly distributed in the co-deposited shell rather than on the outer surface of the material. After etching the silica, the Pt nanoparticles embedded in the shell are exposed, and the catalytic activity of Pt nanoparticles is greatly improved. The above results clearly demonstrate that Pt/NMCT has a high catalytic efficiency in the 4-NP catalytic reduction reaction, and its hierarchical pore structure should be the main reason for the increased activity.

## 4. Conclusions

In summary, this work successfully synthesized Pt/NMCT through the polydopamine-mediated “one-pot” co-deposition process, followed by calcination and etching processes. The loaded Pt nanoparticles had a high loading capacity (9.5 wt%), a small particle size (~2 nm), and a uniform distribution on the carrier. The use of the dual-silica template brought about a hierarchical pore structure with a high specific surface area (620 m^2^ g^−1^) and a large pore volume (1.46 cm^3^ g^−1^). After high-temperature calcination in a nitrogen atmosphere, most of the Pt was reduced and formed ultra-small nanoparticles. In the 4-NP reduction reaction, Pt/NMCT had an apparent rate constant of 0.184 min^−1^, which was significantly superior to SiO_2_ NW@Pt/C-SiO_2_ and Pt/NMCS. This work makes an excellent contribution to the design of supported noble metal catalysts and also highlights the importance of hierarchical pore structures for catalytic activity.

## Data Availability

Not applicable.

## Supplementary Materials

The supporting information can be downloaded at: https://www.mdpi.com/article/10.3390/nano13192633/s1: Figure S1. TEM images of the Pt loaded NMCT synthesized by rotary evaporation and calcination; Figure S2. TEM image of Pt/NMCS.
