# Supplementary material for: One-Pot Synthesis of Ultra-Small Pt Nanoparticles-Loaded Nitrogen-Doped Mesoporous Carbon Nanotube for Efficient Catalytic Reaction
Source: Nanomaterials (Basel). 2023 Sep 25;13(19):2633. doi: 10.3390/nano13192633 (PMC10574567; doi:10.3390/nano13192633)
Supplement: Supplementary file 1 [file nanomaterials-13-02633-s001.zip › nanomaterials-2597800-supplementary.pdf]

Supporting Information

One-Pot Synthesis of Ultra-Small Pt Nanoparticles-Loaded Nitrogen-Doped Mesoporous Carbon Nanotube for Efficient Catalytic Reaction

Qian Zhang [1], Minying Wu [1], Yuanyuan Fang [1], Chao Deng [2], Hsin-Hui Shen [3], Yi Tang [1,*] and Yajun Wang [2,*]

[1] Department of Chemistry, Shanghai Key Laboratory of Molecular Catalysis and Innovative Materials, and Laboratory of Advanced Materials, Fudan University, Shanghai 200438, China; 13110220006@fudan.edu.cn (Q.Z.); 18110220035@fudan.edu.cn (M.W.); 19110220078@fudan.edu.cn (Y.F.)

[2] College of Chemistry & Materials Engineering, Wenzhou University, Wenzhou 325027, China; dengchao@wzu.edu.cn

[3] Department of Materials Science and Engineering, Monash University, Clayton, VIC 3800, Australia; hsin-hui.shen@monash.edu

* Correspondence: authors: yitang@fudan.edu.cn (Y.T.); yajunwang@wzu.edu.cn (Y.W.)

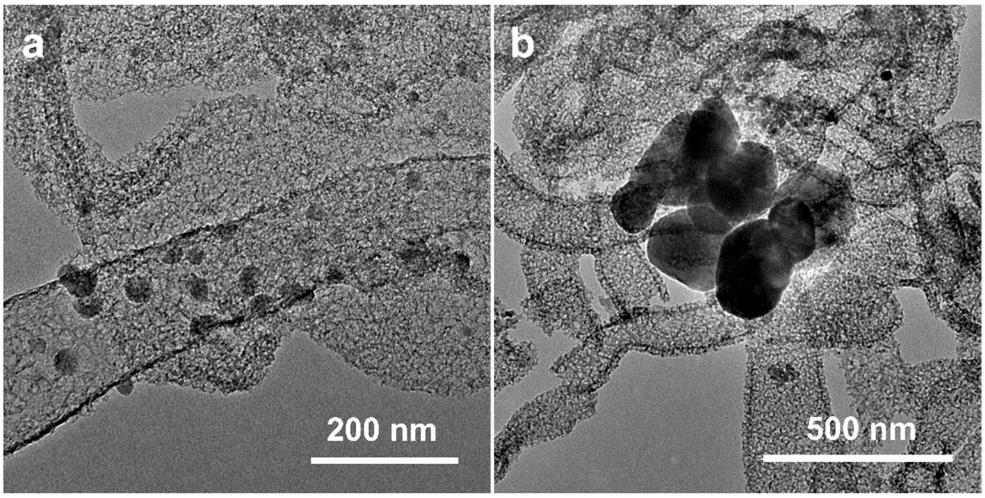

Figure S1. TEM images of the Pt loaded NMCT synthesized by rotary evaporation and calcination.

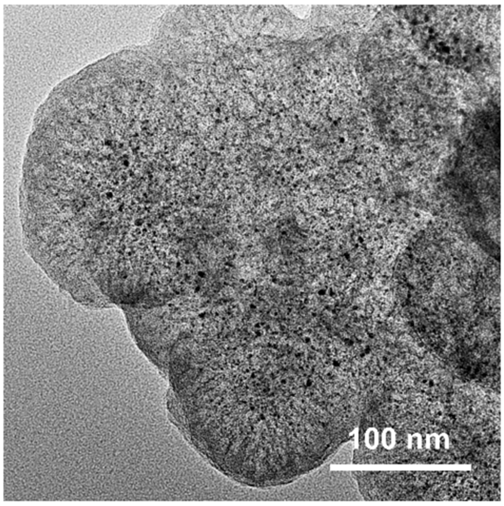

Figure S2. TEM image of Pt/NMCS.
